# Drug–Drug Interactions in People Living With HIV at Risk of Hepatic and Renal Impairment: Current Status and Future Perspectives

**DOI:** 10.1002/jcph.2025

**Published:** 2022-02-08

**Authors:** Nicolas Cottura, Hannah Kinvig, Sandra Grañana‐Castillo, Adam Wood, Marco Siccardi

**Affiliations:** ^1^ Department of Pharmacology and Therapeutics University of Liverpool Liverpool UK

**Keywords:** antiretroviral and in silico modeling, drug–drug interactions, HIV, special populations

## Abstract

Despite the advancement of antiretroviral therapy (ART) for the treatment of human immunodeficiency virus (HIV), drug–drug interactions (DDIs) remain a relevant clinical issue for people living with HIV receiving ART. Antiretroviral (ARV) drugs can be victims and perpetrators of DDIs, and a detailed investigation during drug discovery and development is required to determine whether dose adjustments are necessary or coadministrations are contraindicated. Maintaining therapeutic ARV plasma concentrations is essential for successful ART, and changes resulting from potential DDIs could lead to toxicity, treatment failure, or the emergence of ARV‐resistant HIV. The challenges surrounding DDI management are complex in special populations of people living with HIV, and often lack evidence‐based guidance as a result of their underrepresentation in clinical investigations. Specifically, the prevalence of hepatic and renal impairment in people living with HIV are between five and 10 times greater than in people who are HIV‐negative, with each condition constituting approximately 15% of non‐AIDS‐related mortality. Therapeutic strategies tend to revolve around the treatment of risk factors that lead to hepatic and renal impairment, such as hepatitis C, hepatitis B, hypertension, hyperlipidemia, and diabetes. These strategies result in a diverse range of potential DDIs with ART. The purpose of this review was 2‐fold. First, to summarize current pharmacokinetic DDIs and their mechanisms between ARVs and co‐medications used for the prevention and treatment of hepatic and renal impairment in people living with HIV. Second, to identify existing knowledge gaps surrounding DDIs related to these special populations and suggest areas and techniques to focus upon in future research efforts.

The Joint United Nations Programme on HIV/AIDS (UNAIDS) reported that in 2020 there were approximately 690,000 deaths related to acquired immune deficiency syndrome (AIDS) and 37.6 million people living with human immunodeficiency virus (HIV) globally. Moreover, 27.4 million people were estimated to be accessing antiretroviral therapy (ART).^1^ There are now a multitude of ARVs across several classes available for use in the lifelong treatment of HIV. The World Health Organization provide recommendations for first‐ and second‐line regimens alongside alternative ART strategies for specific scenarios and populations. Current first‐ and second‐line treatments tend to include two nucleoside/tide reverse transcriptase inhibitors and either an integrase inhibitor or a non‐nucleoside/tide reverse transcriptase inhibitor.^2^ Although current treatment strategies involve daily orally administered ARVs, there is increasing interest in the application of long‐acting ARV treatment among clinicians and patients alike.^3^ Long‐acting antiretrovirals (ARVs) have the potential to reduce pill burden and tackle the prominent issues surrounding drug adherence through different technological platforms such as intramuscular injections,^4^ subcutaneous implants,^5^ and microneedle array patches.^6^, ^7^ The first extended‐release long‐acting ARV injectable for HIV treatment, a combination of cabotegravir and rilpivirine, was approved in January 2021 by the US Food and Drug Administration (FDA), demonstrating the potential future of ART.^8^, ^9^ This novel treatment consists of an initial dose of cabotegravir 600 mg and rilpivirine 900 mg, followed by monthly 400 mg of cabotegravir and 600 mg rilpivirine thereafter.^10^

Concomitant treatments in people living with HIV receiving ART, such as those used to treat comorbidities, can lead to polypharmacy, increasing the potential of drug–drug interactions (DDIs).^11^ Furthermore, considering the development of long‐acting ARVs, it is imperative to understand the mechanisms and magnitudes of potential DDIs in this population.^12^ Pharmacokinetic DDIs result from changes in the absorption, distribution, metabolism, and excretion processes of the victim drug, caused by the perpetrator drug, which commonly involve the inhibition or induction of drug‐metabolizing enzymes, transporters, or both. An alteration in the activity or the abundance of these enzymes or transporters could increase or decrease the exposure of the victim drug or the perpetrator drig. Metabolizing enzymes can be divided into phase‐I cytochrome P450 enzymes (CYPs) and phase‐II uridine 5′‐diphospho‐glucuronosyltransferase enzymes (UGTs). Transporters play an important role in the disposition of a drug, specifically in the gastrointestinal tract, liver, and kidneys, although they are located in tissues and organs throughout the body. Transporters can be categorized into two superfamilies: solute carrier (SLC) transporters and ATP‐binding cassette (ABC) transporters.^13^ The changes in plasma concentration resulting from these DDIs can reduce the efficacy and safety of the victim drug. Furthermore, ARV‐resistant mutations can arise from subtherapeutic ARV concentrations, increasing the risk of treatment failure and necessitating alternative ART strategies.

Understanding and assessing people living with HIV is essential for rational and effective therapies. This is of particular relevance in special populations who are often underrepresented in clinical trials and, as a result, lack evidence‐based guidance for their clinical management.^14^, ^15^ Special populations can be characterized by complex physiological changes, and consequently DDI studies conducted in healthy adult volunteers do not always provide a comprehensive description of potential DDIs across different populations. Specifically, DDIs between ART and drugs used for the prevention and treatment of organ impairment can be complex, with a multitude of pharmacological and physiological factors requiring consideration during clinical management. Set upon a backdrop of limited evidence‐based guidance, this can produce unique and challenging scenarios in the clinical setting.

In this review, we present current therapeutic strategies and potential ARV‐related DDIs and their mechanisms involving co‐medications used for the prevention and treatment of hepatic and renal impairment in people living with HIV. In addition, we identify existing knowledge gaps and suggest areas to focus upon in future research efforts to support the clinical management of people living with HIV at risk of hepatic and renal impairment.

## Hepatic Impairment

Liver‐related disease has been estimated to account for 14% to 18% of mortality in people living with HIV and is one of the leading causes of non‐AIDS‐related death, including almost half of deaths among hospitalized people living with HIV.^16^ Although HIV can produce hepatic injury itself, the most common causes of liver disease among people living with HIV are hepatitis C (HCV) and hepatitis B (HBV).^17^ Chronic hepatitis can lead to fibrosis, cirrhosis, and liver failure, with further complications such as ascites, resulting in a 50% 2‐year survival rate.^18^, ^19^ Current therapeutic strategies for hepatic impairment target the stages prior to impairment, such as HCV and HBV infection. These strategies generally consist of multi‐drug regimens, creating multifaceted DDIs in people living with HIV. Although outside the scope of this review, physiological changes caused by hepatic impairment can alter drug pharmacokinetics and DDI magnitudes, with the liver being the main metabolizing organ for the vast majority of small drugs. Hepatic enzyme activity, blood flow, functional liver mass, plasma protein concentration, liver transporter mRNA level, and activity in hepatic impairment conditions have been evaluated;^20^, ^21^, ^22^, ^23^ however, their cumulative impact on varying DDI mechanisms remains unclear. For example, enzyme inhibition has been reported to decrease in people with hepatic impairment, whereas enzyme induction is suggested to remain unchanged.^24^ A previous study compared the area under the curve (AUC) ratio in patients with hepatic impairment and the maximal DDI AUC ratio in healthy patients with the fraction of drug metabolized by CYP3A4. They found a 30% decrease in the AUC ratio in patients with hepatic impairment, compared with healthy patients, for drugs with greater than 50% metabolism via CYP3A4.^25^ However, this study had several limitations and our lack of understanding of these changes highlights the importance of evidence‐based decision making during the clinical management of people living with HIV with hepatic impairment.

### Hepatitis C

**Figure 1:**
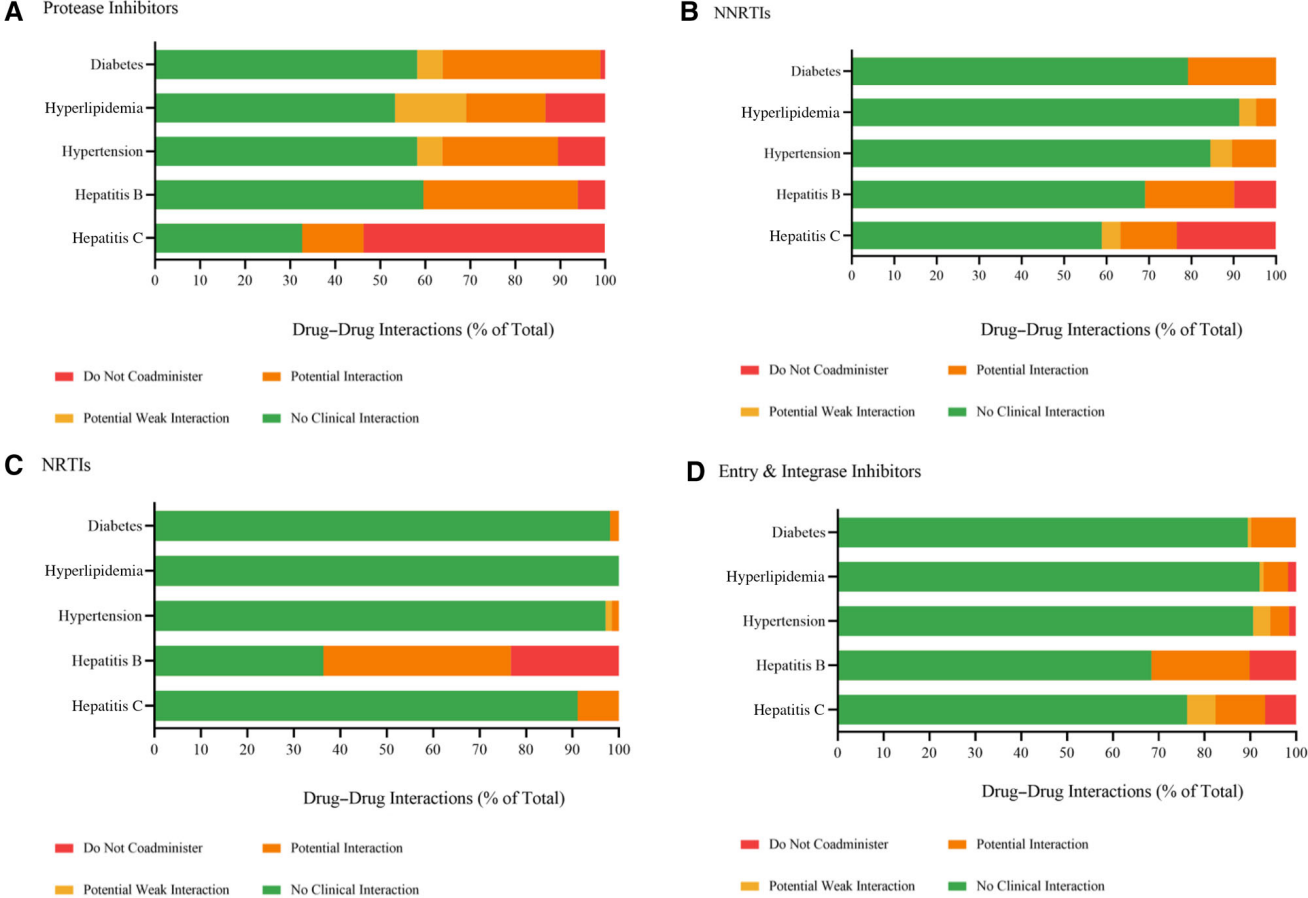
Drug–drug interactions between treatments for diabetes, hyperlipidemia, hypertension, hepatitis B or hepatitis C, and antiretroviral therapy classes: A, protease inhibitor; B, non‐nucleoside/nucleotide reserve transcriptase inhibitor (NNRTI); C, nucleoside/nucleotide reserve transcriptase inhibitor (NRTI); and D, entry and integrase inhibitor (adapted from the HEP Drug Interactions Platform^28^)

Treatment for HCV has evolved from interferon‐based regimens to the currently recommended second‐generation direct‐acting antiviral drugs, and is selected based on virus genotype.^26^ Although recommendations for the treatment of HCV in people living with HIV are the same for those infected with HCV alone, careful consideration must be made regarding potential DDIs between the two treatment strategies.^27^ The Hepatitis Drug Interaction website and HIV Drug Interaction website developed by the University of Liverpool highlighted potential DDIs between commonly administered ARVs and second‐generation direct‐acting antiviral drugs.^28^ As presented in Figure 1, the majority of DDIs that were recommended not to be coadministered with direct‐acting antivirals used for the treatment of HCV involved protease inhibitors, accounting for a total of 53.6% of DDIs. Clinical DDIs were expected to occur between 41.1% of non‐nucleoside reverse transcriptase inhibitors, with 23.3% being recommended not to be coadministered. Entry and integrase inhibitors were found to have 23.8% of DDIs classed as potential weak interaction, potential interaction, and do not coadminister. No clinical interactions were expected with greater than 90% of DDIs between nucleoside reverse transcriptase inhibitors and direct‐acting antivirals.

Sofosbuvir, a nucleotide HCV polymerase inhibitor, presents minimal risk of DDIs with ARVs, mainly through its lack of metabolism by CYP enzymes. Although, as sofosbuvir is a substrate of the efflux permeability glycoprotein (P‐gp) transporter, DDIs are expected between inhibitors and inducers of P‐gp. For example, clinically significant interactions are expected between the less commonly used protease inhibitor (PI) tipranavir because of its inductive potential of P‐gp, reducing the therapeutic efficacy of sofosbuvir.^29^, ^30^, ^31^ Despite no clinically significant interaction, the coadministration of ledipasvir with efavirenz reduced ledipasvir plasma concentrations by approximately 30%. This is thought to occur via induction of CYP3A4 and inhibition of the efflux breast cancer resistance protein (BCRP) transporter.^32^, ^33^, ^34^ In contrast, potential interactions are expected between ledipasvir and tenofovir disoproxil fumarate. Ledipasvir has been shown to increase tenofovir plasma concentrations, posing a risk of nephrotoxicity.^32^ The mechanism of DDI remains unclear as tenofovir is transported by organic anion transporters 1 and 3 (OAT1 and OAT3) and multidrug resistance protein 4 (MRP4), and ledipasvir has not yet been shown to interact with these two transporters.^34^, ^35^ During the administration of the prodrug formulation tenofovir alafenamide there is less tenofovir systemically, and thus the risk of nephrotoxicity and potential DDI with ledipasvir is reduced. This remains true despite the fact that ledipasvir is an inhibitor of P‐gp and BCRP, for which tenofovir alafenamide is a substrate.^34^, ^36^ Similarly to ledipasvir, velpatasvir demonstrates potentially clinically significant interactions with tenofovir disoproxil fumarate, and their coadministration should be carefully monitored. In contrast, velpatasvir undergoes greater metabolism by CYP3A4 and is recommended not to be coadministered with the non‐nucleoside transcriptase inhibitors efavirenz, etravirine, and nevirapine because of their induction characteristics, leading to decreased concentrations of velpatasvir.^32^, ^37^ Of all the sofosbuvir‐containing regimens, the three‐drug combination of sofosbuvir, velpatasvir, and voxilaprevir has the highest risk of potential DDIs with ARVs, as a result of transporter‐based inhibition mechanisms. Sofosbuvir, voxilaprevir, and velpatasvir are substrates of P‐gp and BCRP, with voxilaprevir and velpatasvir also being a substrate of organic anion transporting polypeptides 1B1 and 1B3 (OATP1B1 and OATP1B3).^30^, ^37^, ^38^ Protease inhibitors used for the treatment of HIV are not only inhibitors of CYP enzymes, particularly CYP3A4,^39^, ^40^ but also P‐gp, BCRP, and OATP1B1/OATP1B3 transporters. Specifically, boosted atazanavir and lopinavir should not be coadministered with the combination sofosbuvir, velpatasvir, and voxilaprevir because of an increase in plasma concentrations, although potential DDIs with boosted darunavir are only expected to occur with higher doses.^28^ Daclatasvir is primarily metabolized by CYP3A4 and is a substrate of P‐gp as well as an inhibitor of P‐gp, BRCP, and OATP1B1/OATP1B3. Daclatasvir can therefore incur several potential DDIs with ARVs as both a victim and a perpetrator.^32^, ^41^ However, current guidelines only recommend the use of a reduced daclatasvir dose of 30 mg once‐daily with the protease inhibitor atazanavir to avoid potential toxicity issues.^32^, ^41^, ^42^

The combination regimens that present the greatest risk of potential DDIs with ARVs, specifically non‐nucleoside reverse transcriptase inhibitors and boosted protease inhibitors, are: ombitasvir, ritonavir‐boosted paritaprevir, and dasabuvir; grazoprevir and elbasvir; and glecaprevir and pibrentasvir. First, the combination ombitasvir, ritonavir‐boosted paritaprevir, and dasabuvir are substrates and inhibitors of multiple enzymes and transporters, as summarized in Table 1.^32^ Furthermore, ritonavir is an inducer of CYP1A2, CYP2C8, CYP2C9, CYP2C19, and MRP1.^40^ The complex substrate and inhibitor profile of this regimen presents challenges in identifying the DDI mechanisms involved. Both enzyme and transporter inhibition, as well as enzyme induction, are thought to contribute toward the clinically significant DDIs, and as a result their coadministration is contraindicated. In some instances, the DDI risk arises from the double administration of the protease inhibitor boosters in the fixed‐dose regimens for HIV and HCV, producing unwanted magnitudes of CYP inhibition.^28^ OATP1B1/OATP1B3 transporters also play a major role in the DDIs between paritaprevir and protease inhibitors, with protease inhibitors such as atazanavir reported as strong inhibitors. Additionally, the regimen ombitasvir, ritonavir‐boosted paritaprevir, and dasabuvir should not be coadministered with inducers of CYP3A4, such as non‐nucleoside reverse transcriptase inhibitors, as all four drugs are substrates and could decrease plasma concentrations below the therapeutic window.^28^ Furthermore, increased levels of rilpivirine have been observed when coadministered with this regimen, which could potentially lead to a prolongation of the QT interval.^32^ Drug‐induced QT interval prolongation is a critical issue as it is a precursor for fatal arrythmias such as polymorphic ventricular tachycardia and torsade de pointes.^43^

**Table 1:** Enzyme and Transporter Substrate and Inhibition Profiles of Ombitasvir, Paritaprevir, Dasabuvir, and Ritonavir

Drug	Substrate	Inhibitor
Ombitasvir	P‐gp;^32^, ^127^ CYP3A4;^32^ BCRP^127^	UGT1A1^32^, ^127^; CYP2C8^32^
Paritaprevir	P‐gp and CYP3A4;^32^, ^128^ BCRP, OATP1B1, and OATP1B3^128^	UGT1A1, P‐gp, OATP1B1/3, and BCRP;^32^, ^128^ CYP2C8^32^; and OATP2B1^128^
Dasabuvir	P‐gp and CYP3A4;^32^, ^129^ CYP2C8 and CYP2D6;^32^ BCRP^129^	UGT1A1 and BCRP;^32^, ^129^ and P‐gp (in vitro)^129^
Ritonavir	CYP3A4, CYP2D6, P‐gp, and MRP1^40^	CYP3A4;^32^, ^40^ CYP2D6, P‐gp, MRP1, OATP‐C, and BCRP^40^

BCRP, breast cancer resistance protein; CYP, cytochrome P450; MRP, multidrug resistance protein; OATP, organic anion transporting polypeptides; P‐gp, permeability glycoprotein; UGT, uridine 5′‐diphospho‐glucuronosyltransferase.

The grazoprevir and elbasvir regimen should not be coadministered with inhibitors or inducers of CYP3A enzymes, P‐gp, or OATP1B1/OATP1B3. Furthermore, potential DDIs may occur with substrates of P‐gp, BCRP, and CYP3A4, as elbasvir inhibits both P‐gp and BCRP, whereas grazoprevir inhibits BCRP and is a mild inhibitor of CYP3A4.^32^, ^44^, ^45^ The non‐nucleoside reverse transcriptase inhibitors efavirenz, etravirine, and nevirapine induce CYP3A4, decreasing grazoprevir and elbasvir plasma concentrations below the therapeutic window, resulting in clinically significant DDIs. Protease inhibitors are inhibitors of CYP3A4, P‐gp, and OATP1B1/OATP1B3, and as such they are contraindicated with grazoprevir and elbasvir. Coadministration of glecaprevir and pibrentasvir is not recommended with ARVs that inhibit the P‐gp and BCRP transporters as this may reduce the elimination of both glecaprevir and pibrentasvir, thereby increasing their plasma concentration.^46^, ^47^ A similar effect is expected with the coadministration of glecaprevir and ARVs that inhibit OATP1B1 and OATP1B3.^47^ Additionally, these interactions may increase the risk of alanine transaminase elevations through the significant increase in pibrentasvir plasma concentrations.^48^

### Hepatitis B

The treatment of HBV in people living with HIV involves two nucleoside reverse transcriptase inhibitors, usually lamivudine or emtricitabine together with tenofovir, as tenofovir disoproxil fumarate or tenofovir alafenamide. As these antivirals are also used for the treatment of HIV,^49^, ^50^, ^51^ DDIs can revolve around the potential for “double‐dosing.” Protease inhibitors are mainly contradicted with ARVs used for HBV treatment as they inhibit OATP1B1 and OATP1B3,^52^ for which tenofovir is a substrate, and if tenofovir disoproxil fumarate or tenofovir alafenamide cannot be used safely, the HBV nucleoside analog entecavir, in addition to a fully suppressive ARV regimen, is recommended.^53^ No DDIs are expected with entecavir and any ARVs.^54^ Additionally, as highlighted in Figure 1, nucleoside reverse transcriptase inhibitors have the highest rate of “do not coadminister” recommendations, at 23.4%.

### Liver Disease

The DDIs between ARVs and drugs used to treat complications of liver disease were also explored using the Hepatitis Drug Interaction website and HIV Drug Interaction website, although no clinically significant interactions were found.^54^ In vitro data indicate that furosemide, which can be coadministered with spironolactone to treat ascites, is a weak inhibitor of the renal transporters OAT1 and OAT3, although it is predicted to have a clinically insignificant effect on the OAT1 and OAT3 substrate, tenofovir.^54^

## Renal Impairment

End‐stage renal disease (ESRD) is 2.5‐fold more common in people living with HIV than in the general population.^55^ Similarly, advanced renal impairment disorders such as acute kidney injury (AKI) and chronic kidney disease (CKD) are highly prevalent in people living with HIV, at around 2% and 17%, respectively.^55^ People living with HIV remain at risk of ESRD, AKI, and CKD as a result of the prevalence of risk factors associated with renal impairment.^56^ These risk factors include hypertension, diabetes, and hyperlipidemia, which can affect approximately 35%, 8%, and 35% of people living with HIV, respectively.^57^ As a result, treatment strategies for renal impairment usually revolve around the aforementioned comorbidities. The clinical assessment of potential DDIs between ARVs and commonly used co‐medications in people living with HIV and renal impairment is vital to prevent the development of ESRD, AKI, and CKD, as well as to prevent ineffective ARV treatment.^58^ Although outside the scope of this review, renal impairment has been reported to impact the glomerular filtration rate, affecting overall drug clearance and, in turn, potential DDI magnitudes through changes in perpetrator and/or victim concentrations.^59^ Furthermore, a previous review highlighted the impact of kidney disease on drug metabolism and transport.^60^ Experimental models of ESRD demonstrated decreased activity and downregulation of hepatic and intestinal metabolic enzymes and transporters.^61^ The accumulation of uremic toxins in the blood may contribute either directly by inhibiting enzyme and transporter activity or indirectly by downregulating the transcriptional activation of the gene families via proinflammatory cytokine messengers.^62^, ^63^ This suggests that renal impairment may not only impact DDI magnitude through glomerular filtration rate but also via transporters and metabolism mechanisms, adding to the complexities related to the investigation of DDIs. Similar to the study of hepatic impairment, a previous study compared the AUC ratio in people with renal impairment and the maximal observed DDI AUC ratio in healthy subjects with the fraction excreted unchanged into urine. The AUC ratio for people with renal impairment was greater than in healthy subjects for drugs with limited involvement in hepatic and intestinal enzyme and transporter pathways. Again, this study had several limitations and further clarity on the mechanisms of renal impairment that govern changes in DDI magnitude is required, alongside evidence‐based guidance to aid the clinical management of people living with HIV with renal impairment.^25^

### Hypertension

Hypertension is typically treated using a diverse range of therapeutics, although the preferred frontline therapies in renally impaired populations include angiotensin‐converting enzyme inhibitors, angiotensin receptor blockers, and calcium channel blockers.^64^ As angiotensin‐converting enzyme inhibitors are prodrugs that are not metabolized by CYPs, they are not prone to interacting with ARVs. However, angiotensin receptor blockers are eliminated via hepatic metabolism and/or biliary excretion: some display a higher tendency for interactions with ARVs than angiotensin‐converting enzyme inhibitors as a result of hepatic metabolism via CYP2C9 for losartan, irbersartan, and candesartan.^65^

Similarly, calcium channel blockers primarily undergo CYP450‐mediated metabolism, particularly CYP3A4, and therefore represent the antihypertensive class that interacts most strongly with ARVs.^65^ Significant interactions are thus possible with a broad spectrum of ARVs, notably protease inhibitors and non‐nucleoside reverse transcriptase inhibitors,^65^, ^66^ as highlighted in Figure 1. Interactions resulting from the concurrent inhibition of CYP3A4 from calcium channel blockers and protease inhibitors have the potential to cause serious cardiac effects, such as hypotension, resulting from an increase in calcium channel blocker drug plasma concentration. These cardiac effects have been previously reported for nifedipine extended‐release tablets with ritonavir andindinavir.^67^ Consequently, the clinical monitoring of patients who are taking calcium channel blockers and protease inhibitors is highly recommended to reduce the risk of interactions causing cardiac effects.^68^ It is also worth noting that verapamil is an inhibitor as well as a substrate for both CYP3A4 and P‐gp.^69^

### Type‐2 Diabetes

The treatment of type‐2 diabetes involves both pharmacological and lifestyle strategies, with several key pharmacological agents, including biguanides (eg, metformin, sulfonylureas, and thiazolidines).^70^ Metformin is currently the first‐line treatment for type‐2 diabetes. With its lack of hepatic‐mediated metabolism, interactions resulting from induction and inhibition of hepatic enzymes, such as CYP450, are not relevant for altering metformin concentrations. However, metformin is susceptible to interactions resulting from the inhibition of transporters associated with metformin uptake and secretion in the liver, kidney, and skeletal muscle, notably organic cation transporters (OCTs) and multidrug and toxin extrusion proteins (MATEs).^71^, ^72^ Dolutegravir is responsible for the inhibition of transporters, including the renal transporter OCT2,^73^ and has been shown to cause significantly increased metformin exposure by reducing its renal clearance and tubular secretion.^72^ The concomitant use of metformin and dolutegravir is not associated with a higher risk for hypoglycemia in healthy populations; however, dose adjustment may be required at the start or termination of dolutegravir therapy when coadministered with metformin to maintain control of glycaemia.^72^, ^74^ Furthermore, as metformin is excreted via the kidneys, people with renal impariment have a higher risk of lactic acidosis from metformin toxicity.^72^, ^75^ Additionally, metformin is susceptible to interactions with the protease inhibitor‐boosting agent cobicistat, which reversibly inhibits MATE‐1, an efflux transporter essential for metformin clearance.^76^ As a result, metformin concentrations increase as a result of reduced renal elimination and dose adjustments are therefore recommended.^76^

Glibenclamide, like the majority of the sulfonylurea family, is primarily metabolized by CYP2C9 and is liable to potential interactions with ARVs.^77^ These include, but are not limited to, CYP2C9 inhibition by the non‐nucleoside reverse transcriptase inhibitors etravirine and efavirenz and induction by the protease inhibitor ritonavir or integrase strand transfer inhibitor elvitegravir. Note that the inhibition of CYP2C9 via non‐nucleoside reverse transcriptase inhibitors may increase the risk of hypoglycemia.^78^, ^79^

### Hyperlipidemia

Statins are 3‐hydroxy‐3‐methylglutaryl coenzyme A (HMG‐CoA) reductase inhibitors and represent the frontline lipid‐lowering agents for the treatment of hyperlipidemia. Although fibrates, as well as other lipid‐modifying drugs such as ezetimibe, can be used. Several statins are substrates of CYP450 enzymes and are subject to clinically significant DDIs with strong CYP450 inhibitors and inducers. Lovastatin and simvastatin show the highest sensitivity to CYP3A4 inhibition, which has been associated with fatal cases of rhabdomyolysis.^80^ Lovastatin and simvastatin exposures are expected to be decreased through CYP induction when coadministered with efavirenz, etravirine, or nevirapine, which may require a dosage adjustment or close monitoring.^54^ In contrast, rosuvastatin, pravastatin, and pitavastatin undergo minimal metabolism and are transported by P‐gp and OATP1B1/OATP1B3; however, their coadministration with strong inhibitors of these transporters may cause clinically significant DDIs. Specifically, the inhibition of OATP1B1 and OATP1B3 can reduce the effectiveness of treatment as they are responsible for transport into the liver to the sites of statin action.^81^ As a result, statins avoiding CYP450 metabolism are favored in people living with HIV on protease inhibitor‐containing regimens because of their inhibitory properties, although transporter‐based DDIs with protease inhibitors must also be taken into consideration during treatment selection.^54^, ^81^, ^82^ For example, the coadministration of rosuvastatin with ritonavir‐boosted lopinavir increased the rosuvastatin AUC and peak plasma concentration (*C*_max_) by 2.1‐ and 4.7‐fold, respectively.^83^ It is therefore recommended to not exceed 10 mg of rosuvastatin per day and to monitor for side effects.^54^

Fibrates also represent an invaluable lipid‐lowering agent and include drugs such as bezafibrate, clofibrate, fenofibrate, and gemfibrozil. Gemfibrozil undergoes primarily hepatic metabolism via UGT2B7 and has been associated with reduced exposure of gemfibrozil when coadministered with ritonavir‐boosted lopinavir in people living with HIV, potentially as a result of a decrease in absorption or induction of glucuronidation. However, the underpinning mechanism behind this interaction remains unknown.^84^, ^85^ As a result of its weak interaction, no dose adjustment is recommended when prescribing these agents.^85^ Alternatively, fenofibrate does not display an interaction with ritonavir‐boosted lopinavir.^86^ Ezetimibe metabolism is primarily UGT related, notably UGT1A1, UGT1A3, and UGT2B15,^87^ and transported by OATP1B1.^88^ The interaction between ezetimibe and atazanavir has not yet been studied; however, atazanavir is a known inhibitor of UGT1A1 as well as the liver transporter OATP1B1, thus an interaction between these two drugs is expected. Close monitoring is recommended.^89^

In conclusion, as summarized in Figure 1, more than 80% of the DDIs between ARVs, classed as non‐nucleoside reverse transcriptase inhibitors, nucleoside reverse transcriptase inhibitors, and entry and integrase inhibitors, and drugs used for the treatment of diabetes, hypertension, and hyperlipidemia were expected to have no clinical interaction. In contrast, approximately 40% of the DDIs between protease inhibitors and drugs used for the treatment of diabetes, hypertension, and hyperlipidemia were classed as potential weak interaction, potential interaction, or do not coadminister.

## Future Perspectives

Historically, potential risks and logistical barriers related to special populations have resulted in their omission from clinical trials. However, recent efforts to increase awareness of the resulting risks of their neglect and the importance of obtaining evidence‐based guidance from the affected populations aim to challenge current practices.^14^, ^90^ Meanwhile, the need for alternative approaches to fill the knowledge gap during the investigation of DDIs is imperative to improve the clinical management of special populations. This is of particular relevance in people living with HIV considering the rise in those aged ≥65 years who have an increased risk of comorbidities, specifically those related to hepatic and renal impairment, and potential DDIs as a result of polypharmacy.^11^, ^91^, ^92^ Both in vitro and in silico techniques are employed for the assessment of DDIs, with the FDA and European Medicine Agency providing guidance on their implementation.^93^, ^94^, ^95^ A recent analysis of physiologically based pharmacokinetic (PBPK) modeling applications reported 18% and 22% of published models involved special populations and DDIs, respectively, with models in special populations more than doubling over the past 20 years.^96^ The FDA reported almost a 100‐fold increase in drug approval packages containing PBPK analyses from 2008 to 2017, with 60% regarding DDIs.^97^ However, challenges in current in vitro–in vivo extrapolation (IVIVE) techniques as well as limited knowledge of the physiological changes in special populations and lack of clinical data available for method verification continue to impede the successful application of these techniques during the drug development process.^14^, ^15^, ^98^

As discussed, in vitro studies are utilized to investigate the metabolic and transporter pathways of a drug and its potential DDIs through the application of known victim and perpetrator drugs.^94^ The resulting data can be extrapolated from in vitro to in vivo through specific IVIVE equations and in silico techniques, such as PBPK modeling.^93^, ^94^, ^99^, ^100^ Currently, enzyme‐mediated DDI in vitro studies are more established than transporter‐mediated DDI in vitro studies because of the limited knowledge surrounding transporters and the greater complexities related to data extrapolation.^94^, ^101^, ^102^, ^103^ However, neither have comprehensive methodologies for their in vitro investigation in special populations, generating a gap in the required knowledge and integration through in silico applications. There are several factors hindering the in vitro assessment of DDIs in special populations, including ethical, logistical, physiological, technological, and knowledge‐based limitations. In the example of hepatic and renal impairment, in vitro strategies commonly use primary human liver and kidney cells to mimic impaired states, although the accurate prediction of the observed effects in vivo is poor as a result of the inability of the system to replicate and sustain the microenvironment of the organ. The continued development and application of organ‐on‐a‐chip in vitro technologies could help mitigate some of the abovementioned challenges, providing a sustainable resource of data required to produce evidence‐based guidance for special populations.^104^, ^105^, ^106^, ^107^

More specifically, PBPK modeling utilizes mathematical equations to describe the physiological characteristics and absorption, distribution, metabolism, and excretion (ADME) processes of the human body alongside the physicochemical properties of a drug to predict drug plasma concentrations over time in a cohort of virtual patients. PBPK models are developed and verified using in vitro and clinical data, and can be tailored to predict a variety of clinical scenarios including but not limited to DDIs, special populations, drug development, and administration technologies.^96^, ^108^ This modeling approach provides an ethical and viable alternative to clinical trials, providing predicted data to aid in clinical management. However, caution must be taken when analyzing such data and a clear understanding of the limitations of the PBPK model must be taken into consideration. Generally, limitations encompass the general understanding of the physiological and ADME processes of the simulated population and drugs, as well as the quality and quantity of in vitro and clinical data used for model development and verification. Thus far, few PBPK models for the prediction of drug pharmacokinetics in patients with hepatic impairment^109^ and in patients with renal impairment^110^, ^111^, ^112^ have been developed, and to our knowledge only one PBPK model has assessed DDIs in the presence of organ impairment.^113^ This model investigated the DDI between quinine and ritonavir‐boosted lopinavir in patients with chronic renal failure and in patients with mild, moderate, and severe hepatic insufficiency.^113^ To maintain quinine concentrations within the therapeutic window, a dose adjustment from 1800 mg t.i.d. in healthy patients to 647 mg b.i.d in patients with chronic renal failure, 648 mg t.i.d in patients with mild and moderate hepatic insufficiency, and 324 mg b.i.d in patients with severe hepatic insufficiency was predicted.^113^ In 2021, 19 member companies of the International Consortium for Innovation and Quality in Pharmaceutical Development wrote a white paper detailing PBPK simulations of 29 compounds with 106 organ impairment study arms: 50 renal impairment and 56 hepatic impairment arms. The PBPK models predicted >90% and >70% of the AUC ratios of patients with renal impairment and patients with hepatic impairment versus healthy patients within 2‐fold of the observed clinical data, respectively.^114^ The study demonstrates improved awareness of the issues surrounding pharmacokinetics in special populations with organ impairment and the capability of PBPK modeling to help fill the knowledge gap, even if DDI predictions remain missing from those studies. To note, no PBPK models have been generated for the simulation of drug pharmacokinetics in people living with HIV, although the effect of HIV on drug pharmacokinetics is currently unclear.^115^

Additionally, with the implementation of long‐acting ARV treatment strategies to reduce the pill burden and improve adherence in people living with HIV, it is important to understand their impact on DDIs. With some long‐acting applications unable to be removed once administered, we must have clarity on potential DDIs as any required changes may not be actionable on a short‐term basis. PBPK modeling has been employed to investigate the magnitude of DDI between long‐acting cabotegravir and rilpivirine and the antituberculosis drug, rifampicin. Their co‐medication was predicted to generate subtherapeutic concentrations, which is similar to the DDI between the oral formulations of cabotegravir and rilpivirine with rifampicin.^116^, ^117^ In addition, a review was recently published describing potential DDIs with long‐acting cabotegravir and rilpivirine and highlighted that although intramuscular administration reduces the DDI potential in the gut, hepatic DDIs involving UGT1A1 and CYP3A4 are still relevant, respectively.^12^ Questions surrounding long‐acting DDIs extend to special populations, whereby their impact is yet unclear and further research is required to provide evidence‐based guidance. Interestingly, a PBPK model describing the pharmacokinetics of long‐acting cabotegravir and rilpivirine in patients with hepatic impairment and Child–Pugh scores of A, B, and C predicted that no dose alteration would be required in this population.^118^, ^119^ Moreover, to our knowledge, no PBPK models for the investigation of long‐acting ARV DDIs in patients with either hepatic or renal impairment have been created. Expanding future research efforts in PBPK modeling of DDIs in special populations, as well as improving our current understanding of special population physiology and mechanisms of DDIs, will enhance the reliability of in silico strategies in drug development and clinical management. For example, rifampicin is a strong inhibitor and inducer of several transporters and enzymes, and extrapolating the DDIs of mild‐to‐moderate inhibitors and inducers from healthy patients to patients with hepatic impairment may not be reliable.

Diverting from mechanistic approaches, novel mathematical techniques such as artificial intelligence and machine learning methods ranging from regression analysis to deep learning and neural networks have recently been utilized for the investigation of DDIs as well as in the prediction of ADME properties and adverse effects.^120^, ^121^, ^122^, ^123^, ^124^, ^125^ Although the application of these techniques for the prediction of DDIs is in its infancy, mainly focusing on CYP‐mediated processes, they are slowly gaining traction as powerful pharmacological tools. To date, there have been no computational algorithms used for the assessment of potential DDIs in hepatic or renal impairment, or any other special populations, for that matter. However, it is apparent that novel mathematical techniques such as these have great potential to fill the knowledge gap in underrepresented populations and clinical scenarios, and future research initiatives should encompass these techniques. For example, there are currently several new agents in development for the treatment of chronic HBV with mechanisms involving targeting viral entry, covalently closed circular DNA (cccDNA), viral transcripts, core protein assembly modulators, and hepatitis B surface antigen (HBsAg) release inhibitors. The role of these new treatment strategies in people living with HIV coinfected with HBV is unclear, and clinical trials in these special populations are not always possible; however, potential DDIs could be investigated through the application of the in silico tools described above.^126^

## Conclusion

Despite advancements in ART DDIs remain a major concern in people living with HIV, and numerous research initiatives have taken place to develop our fundamental understanding of DDIs and improve upon strategies implemented for their clinical management. However, special populations remain underrepresented in such initiatives, particularly in clinical trials, resulting in a lack of evidence‐based guidance for safe and effective treatments. This review summarized potential DDIs and their mechanisms in people living with HIV at risk of hepatic and renal impairment, and highlighted areas to focus upon in future research efforts. Specifically, the inclusion of these special populations in clinical investigations is paramount, and where inclusion is unattainable the development and application of in silico techniques to provide simulated evidence‐based guidance is essential. Moreover, the remaining knowledge gaps surrounding the mechanisms of DDIs in hepatic‐ or renal‐impaired conditions require further research with in silico techniques, such as PBPK modeling, artificial intelligence, machine learning, and algorithm‐based approaches, providing unique opportunities for this endeavor. A special focus might be initiated on already known potential interactions or on drug combinations with pharmacokinetic pathways that are likely to be impacted in either hepatic‐ or renal‐impaired conditions.
